# Examining the Hospital Elder Life Program in a rehabilitation setting: a pilot feasibility study

**DOI:** 10.1186/s12877-016-0313-3

**Published:** 2016-07-18

**Authors:** Kelsey Huson, Paul Stolee, Nancy Pearce, Corrie Bradfield, George A. Heckman

**Affiliations:** School of Public Health and Health Systems, University of Waterloo, Waterloo, ON N2L 3G1 Canada; School of Health and Life Sciences and Community Services, Conestoga College Institute of Technology and Advanced Learning, Kitchener, ON N2G 4M4 Canada; Grand River Hospital, Kitchener, ON N2G 1G3 Canada; Schlegel-UW Research Institute for Aging, Waterloo, ON N2J 0E2 Canada

**Keywords:** Hospital Elder Life Program, Rehabilitation, Delirium, Evaluation

## Abstract

**Background:**

The Hospital Elder Life Program (HELP) has been shown to effectively prevent delirium and functional decline in older patients in acute care, but has not been examined in a rehabilitation setting. This pilot study examined potential successes and implementation factors of the HELP in a post-acute rehabilitation hospital setting.

**Methods:**

A mixed methods (quantitative and qualitative) evaluation, incorporating a repeated measures design, was used. A total of 100 patients were enrolled; 58 on the pilot intervention unit and 42 on a usual care unit. Group comparisons were made using change scores (pre-post intervention) on outcome measures between pilot unit patients and usual care patients (separate analyses compared usual care patients with pilot unit patients who did or did not receive the HELP). Qualitative data were collected using focus group and individual interviews, and analyzed using emergent coding procedures.

**Results:**

Delirium prevalence reduced from 10.9 % (*n* = 6) to 2.5 % (*n* = 1) in the intervention group, while remaining the same in the usual care group (2.5 % at both measurement points). Those who received the HELP showed greater improvement on cognitive and functional outcomes, particularly short-term memory and recall, and a shorter average length of stay than patients who did not. Participant groups discussed perceived barriers, benefits, and recommendations for further implementation of the HELP in a rehabilitation setting.

**Conclusions:**

This study adds to the limited research on delirium and the effectiveness of the HELP in post-acute rehabilitation settings. The HELP was found to be feasible and have potential benefits for reduced delirium and improved outcomes among rehabilitation patients.

## Background

Delirium is common among older adults in health care settings [1, 2], and is consistently associated with increased rates of morbidity, mortality, long-term care placement and longer, costlier hospitalizations [1, 3–8]. Delirium can be prevented [2, 5, 9–11]; evidence shows that primary prevention is the most effective strategy to reduce the incidence of inpatient delirium, decrease length of stay, and enhance functional recovery [4, 6, 8, 12].

The Hospital Elder Life Program (HELP) is a multicomponent intervention to prevent delirium and functional decline in hospitalized older adults [13–15]. It offers practical interventions that target six risk factors for delirium, including an orientation protocol targeting cognitive impairment, a sleep protocol to promote sleep enhancement, early mobilization and minimum restraints to prevent deconditioning, adaptive equipment and aids for vision optimization, wax removal and aids for the hearing impaired, and attention to nutrition and hydration. Implemented by an interdisciplinary staff and trained volunteers within existing hospital units, the HELP does not require a specialized geriatric unit [5, 13, 15, 16].

The HELP has been shown to prevent delirium, cognitive and functional decline, and other common geriatric complications of hospitalization [13]. Studies have also shown the program to be effective in improving quality of care, enhancing patient [13], family and nursing satisfaction with care [17], and reducing length of stay [14, 15]. The HELP has been disseminated on medical, geriatric, and surgical units [14, 18–21]. To date, the HELP had not been examined in a rehabilitation hospital setting.

Following a consultation process with various health care providers [22], the HELP was implemented in two hospitals in the Waterloo Region of Ontario, Canada, including in a Restorative Care program. Restorative Care is a slow-stream, general rehabilitation program with 59 beds on two separate units, one with 32 beds and one with 27 beds.

The purpose of this pilot study was to examine potential successes and barriers to the implementation of the HELP in a post-acute rehabilitation hospital setting. Specifically, this study sought to determine 1) if changes in scores (pre-post treatment) on measures of functional and cognitive outcomes differ between patients who received the HELP and those who did not; and 2) patient, caregiver, volunteer, and staff perceptions of, and satisfaction with, the HELP. Information from this study could be used to design future, larger-scale studies.

## Methods

**Fig. 1:**
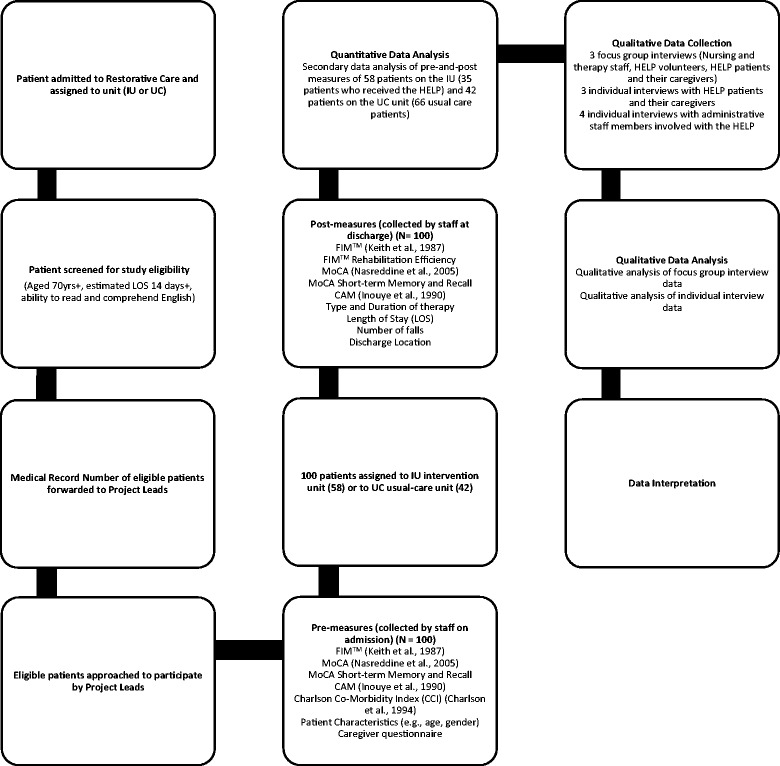
Flow chart of study procedures

Random assignment of subjects was not feasible for this study. Patients were assigned to each unit based on bed availability, reducing the risk of bias. The two units were comparable in both size and patient population. We chose the 32-bed unit as the intervention unit (IU). Patients on the 27-bed unit received usual care (UC). It was thus possible to conduct a comparison between similar patients who received the HELP or received usual care. The processes of recruitment, data collection, data analysis, and data interpretation are illustrated in Fig. 1.

### Sampling

#### Patients

Patients admitted to the Restorative Care program from September 2013 to June 2014 who satisfied the eligibility criteria of the HELP were screened for enrollment. Patients were 70 years of age and older, able to read and comprehend English, with a minimum expected length of stay of 14 days to allow for a sufficient number of interventions to demonstrate change. A non-probability consecutive sampling strategy was used. Patients were screened for eligibility by the Resource Nurse at admission. The Medical Record Number of eligible patients was forwarded to the Elder Life Specialist (ELS), who then approached patients and their caregivers to explain the study. Eligible patients were enrolled in the HELP notwithstanding their participation in the research project.

The sample size was first estimated based on the original study by Inouye and colleagues [5] that examined the effectiveness of the HELP in an acute care hospital. The investigators found that the incidence of delirium in the intervention group was 9.9 % compared to 15 % in the control group [5]. Using these proportions as estimates and with alpha = 0.05 (95 % confidence) and beta = 0.2 (80 % power), a sample size of 686 per group would be required [23]. A sample size of this magnitude was not feasible for this study.

Another outcome of interest in this study was functional recovery. This was measured using the Functional Independence Measure (FIM™) [24]. To demonstrate an estimated clinically important difference of six points in FIM™ scores between groups (IU patients and UC patients) with an estimated standard deviation of 10, and again with alpha = 0.05 and beta = 0.2, a sample size of 47 per group was targeted [23].

A total of 100 patients were enrolled in the study, 58 on unit IU and 42 on unit UC. Only 35 of the 58 patients on unit IU received the HELP. The only reason for attrition was death (*n* = 4); complete information was not available for these subjects, and they were not included in the study.

Purposeful sampling was used to collect qualitative data. To recruit patients for the focus group interview, posters were placed throughout the intervention unit (IU). Project leads provided interested patients with further information. Six patients participated in the interviews; three attended a scheduled focus group interview, and three participated in individual interviews. Five of the six participants were female.

#### Caregivers

Caregivers of patients who participated in the HELP were asked to complete a self-reported questionnaire and to be interviewed. Return of the completed self-report questionnaire was indicated as permission to use responses in the study. Caregivers were excluded if they were unable to read, write, and/or comprehend English. A total of 87 caregivers completed the self-reported questionnaires, 60 of whom cared for patients who received the HELP and 27 of whom cared for patients who received usual care.

Caregivers were recruited for the focus group interview together with the patients they cared for. Five caregivers (three female) of patients who participated in the HELP were interviewed. Four caregivers attended interviews together with the patients they cared for, and one was interviewed individually. Three were spousal caregivers, and two were siblings of patients who participated in the HELP.

#### Volunteers

HELP volunteers involved with the program for a minimum of one month were asked to participate in a focus group interview. Volunteers were excluded if they had not been involved with the HELP for at least one month to ensure sufficient information on their experience with the program. A total of three HELP volunteers (mean age 19; two female) attended the scheduled focus group interview.

#### Staff

Staff members who were involved with the HELP for at least one month (to ensure sufficient experience with the program) were eligible to participate in an interview. Thirteen staff members (11 females) participated in the interviews; nine nursing and therapy staff members attended the scheduled focus group interview, and four administrative staff members participated in one-on-one interviews. Participants consisted of nursing, therapy, and administrative staff members who were involved in the implementation of the HELP. The average age of participating staff members was 40 years.

This study was reviewed and received ethical clearance by both the Tri-Hospital Research Ethics Board (#2012-0496) and the University of Waterloo Office Of Research Ethics (ORE #19900). Written informed consent was obtained from all participants.

### Intervention and usual care

The HELP was modified to be relevant for patients on a rehabilitation unit and was implemented by specially trained volunteers. Volunteers completed a minimum of twelve hours of in-class training, which included a volunteer manual, education, hands-on experience and shadowing. The modified program consisted of two HELP interventions scheduled daily, rather than the standard three, because patients were participating in regular rehabilitation therapy as part of usual care on the Restorative Care unit. The sleep protocol was limited to the provision of a warm blanket because a sufficient number of HELP volunteers were not available during evening hours. The early mobility protocol was limited due to the restrictive weight bearing status of most patients, and leg extensions were added to the range-of-motion activities to be performed while sitting in a wheelchair. Assistance at meals was limited to set-up and encouragement, as patients could self-feed and dined communally.

Usual care consisted of routine hospital care provided by clinical staff on the Restorative Care unit. The HELP volunteers did not provide the program interventions to patients on the usual care unit and clinical staff who worked on UC did not provide care to patients on the intervention unit, minimizing contamination and co-intervention.

### Outcomes

The primary outcomes of this study were the changes in cognitive and functional status between admission and hospital discharge. Patient outcome measures and self-reported caregiver questionnaires were collected by hospital staff. Cognitive function was measured using the Montreal Cognitive Assessment (MoCA) [25], a 30-item screening tool for dementia and mild cognitive impairment that assesses visuospatial function, short-term memory, attention, recall, and working memory [26]. Short-term memory and recall was assessed as a separate outcome measure using these domains of the MoCA.

Functional status was measured using the Functional Independence Measure (FIM™) [24], an 18-item standardized assessment of motor function (13 items) and cognition (five items). FIM™ items are scored on a 7-point scale from 7 (independent) to 1 (dependent) with an overall maximum score of 126. The MoCA was collected by the ELS; the FIM™ was collected by Occupational Therapy (OT) or Physical Therapy (PT) staff and the ELS. Rehabilitation efficiency was also assessed by dividing the change in FIM™ scores from admission to discharge by the length of stay.

The Confusion Assessment Method (CAM) [27] was used to screen for delirium, and was collected by the nursing staff. The CAM was derived from the DSM-III-R criteria for delirium including acute changes in mental status, fluctuating course, inattention, disorganized thinking, psychomotor agitation or retardation and altered level of consciousness. The CAM is used routinely by clinicians to quickly and accurately detect delirium.

Secondary outcomes included comorbidity, number of falls, type and duration of therapy, discharge location, and length of stay. The Charlson Co-morbidity Index (CCI) [28] was used to assess co-morbidity at admission by predicting 1-year mortality based on the presence or absence of 22 conditions according to their relative risk of death and patients’ current age. Falls were recorded by nurses in the Patient and Visitor Safety Reporting System (RISKPRO) as per the hospital’s Post Fall Management Policy. Amount of time spent in therapy, frequency of therapy sessions and type of therapy (OT, PT) were recorded by therapy staff. Discharge location and length of stay were extracted from the Electronic Medical Records (EMR) by project leads.

To describe the sample, patient characteristics (e.g., age, gender, admitting diagnosis) were also collected. Data were then recorded in the hospital’s EMR. To describe the caregiver sample, self-reported questionnaires were collected. The self-reported questionnaire was developed specific to this study to gather information on caregiver characteristics (e.g., gender) [29], kin relationship [29], geographic distance from care recipient [30], co-residence [29], and caregiver self-reported health [31, 32] that are known to predict placement in long-term care. Each outcome measure was chosen with consideration to its feasibility, acceptability and appropriateness for quantifying relevant outcomes.

### Qualitative measures

Qualitative methods were used to gain an in-depth understanding of staff, volunteer, patient and family perceptions of, and satisfaction with, the HELP. To encourage a variety of perspectives, focus group interviews were chosen following procedures recommended by Krueger and Casey [33]. Smaller sized focus groups and shorter sessions (under 60 min) were considered more realistic for the older patient population and their caregivers, as well as busy staff members and volunteers. All focus group interviews took place at the study site. The facilitator was accompanied by the same recorder for each focus group. The recorder developed a seating plan to identify participant location and assisted in taking field notes while the facilitator led the discussion. Pseudonyms were used in the transcripts to identify speakers. All participants provided written informed consent, and all focus group interviews were audiotaped. Participants were asked to complete a brief background questionnaire to describe the sample. At the end of the interview, the facilitator offered participants the opportunity to provide a final comment.

Individual interviews were conducted with administrative staff members involved with the HELP. Issues of acceptability, feasibility and sustainability were examined following a semi-structured interview guide. Interviews were scheduled to take place at the study site. The interviews were brief (approx. 10–20 min). Consent was obtained for each interview to be audiotaped. In addition, three individual interviews were conducted with patients and caregivers who were unable to attend the scheduled focus group interview. The same procedures were followed as mentioned above.

### Data analysis

Quantitative and qualitative methods were considered equal [34, 35]. During the initial analysis, quantitative and qualitative data were analyzed separately. A triangulation approach [36] was used by merging the results of various data sources (patient outcomes, self-reported questionnaire, and focus group and individual interviews) to cross-validate the study findings [11, 37]. Comparisons were made examining similarities and differences in the results from the two data types [11, 34].

Quantitative data analysis was conducted using the software package IBM SPSS Statistics version 22 [38]. Data were de-identified by assigning participants numbers. Frequency and percent distributions were reported for all categorical demographic variables; the range, mean, and standard deviation were reported for all continuous data. Group comparisons of variables were investigated using appropriate statistics. Chi-square tests were used for categorical variables. T-tests were used for continuous variables to compare the means of the two groups. To examine differences in outcomes between patients who received the HELP and those who did not, change scores were calculated for each measure by subtracting the baseline score from the follow-up score.

Non-paired t-tests were conducted to determine differences in outcomes between patients on IU and patients on UC. In addition, non-paired t-tests were conducted to compare differences in outcomes between patients who received the HELP and those who did not. The means of the change scores were used to compare the two groups. *P*-values of less than or equal to 0.05 were considered statistically significant.

Qualitative data were analyzed using recommended procedures [39–41]. Emergent coding was used to manually develop codes. Emerging and relevant codes were highlighted using a distinct color for each category. Text highlighted in the same color was grouped together to sort and organize the data, creating themes [42]. The data were subsequently reviewed to ensure that the codes were well-grounded to fit the data. Each interview was analyzed individually before comparisons were made with the other interviews. Themes within a particular interview were examined before exploring those that emerged across groups. Similarities and differences in themes that emerged among the interviews were examined. Member checking was undertaken by e-mailing participants who provided their contact information. Three staff participants responded, and all respondents verified the findings. Efforts to ensure methodological rigor were employed through auditing and regular consultations with colleagues.

## Results

### Patients

Comparisons between patients were examined in two ways: 1) patients on IU (*n* = 58) versus patients on UC (*n* = 42); 2) patients who received the HELP (*n* = 35) versus patients who did not receive the HELP (*n* = 66). Patients who received the HELP (intervention group) did not differ significantly in any of the characteristics at admission compared to those who did not (usual care group). The primary admission diagnosis for both groups (50 % of admissions) was fracture. Scores on the CCI were similar, with an average of 2.1 and 2.0 for the intervention and usual care groups, respectively. The total amount of therapy (physiotherapy, occupational therapy and speech language pathology) was similar between the patient groups. Average lengths of stay were 52.3 days and 59.2 days for the intervention and usual care groups, respectively. The majority of patients were discharged home with assistance in both the intervention group and the usual care group (33 % vs. 34 %) or to a retirement residence with assistance (28 % vs. 27 %). The baseline characteristics of the patients of each group, according to unit, are shown below in Table 1.

**Table 1:** Characteristics of the patients, according to study group (by unit)

Characteristic	Intervention group^*, **^	Usual care group^*, **^
	(*N* = 58)	(*N* = 42)
Age – yr		
Mean ± SD	82.7 ± 7.9	82.7 ± 9.0
Gender – no. (%)		
Female	35 (60)	27 (64)
Male	23 (40)	15 (36)
Pre-Admission Housing – no. (%)		
Own home	40 (69)	25 (60)
With relatives (not spouse) in relative’s home	2 (3)	4 (10)
Retirement	16 (28)	13 (31)
Received HELP Prior – no. (%)		
Yes	8 (14)	9 (21)
No	51 (86)	33 (79)
Falls Prior – no. (%)		
.00	18 (31)	11 (27)
1.00	30 (52)	27 (66)
2.00	2 (3)	1 (2)
3.00	8 (14)	2 (5)
Admitting Diagnosis – no. (%)		
Fracture	29 (50)	21 (50)
Neoplasms/Nervous System	3 (5)	2 (5)
Genitourinary System	2 (3)	1 (2)
Respiratory System	1 (2)	0
Digestive System	2 (3)	2 (5)
Musculoskeletal System and Connective Tissue	3 (5)	2 (5)
Endocrine, Nutritional, Metabolic/Circulatory System	4 (6)	1 (2)
Skin and Subcutaneous Tissue	3 (5)	3 (7)
Other	10 (17)	9 (21)
CCI		
Mean score ± SD	2.1 ± 2.1	2.0 ± 1.9
Total Amount of Therapy - no. of sessions		
PT		
Mean ± SD	33.2 ± 15.9	27.6 ± 15.8
OT		
Mean ± SD	24.9 ± 10.9	26.7 ± 15.5
SLP		
Mean ± SD	0.5 ± 1.3	0.2 ± 0.7
Discharge Location – no. (%)		
Own home	3 (5)	2 (5)
Home with relative (not spouse) in relative’s home	2 (3)	2 (5)
Retirement home	4 (7)	2 (5)
Retirement home with home care	16 (28)	11 (27)
Nursing home	4 (7)	2 (5)
Home with home care	19 (33)	14 (34)
Home awaiting long-term care	2 (3)	1 (2)
Other	8 (14)	7 (17)

*Plus-minus values are means ± SD

**Percentages were rounded to the nearest whole number

### Caregivers

The caregiver groups did not differ significantly in their baseline characteristics. Caregivers of patients in the intervention group were mostly daughters (38 %) and spouses (33 %); daughters (56 %) were the primary caregivers of most patients in the usual care group. Most caregivers of patients in the intervention group (59 %) and in the usual care group (44 %) rated their health as good; with fewer caregivers of patients in the intervention group rating their health as excellent compared to those caring for patients in the usual care group (23 % vs. 40 %). The baseline characteristics of the caregivers, according to patient unit, are shown in Table 2.

**Table 2:** Characteristics of the caregivers, according to study group (by unit)

Characteristic	Intervention group^*, **^	Usual care group^*, **^
	(*N* = 60)	(*N* = 27)
Age – yr		
Mean ± SD	62.1 ± 14.9	60.8 ± 9.3
Gender – no. (%)		
Female	42 (74)	22 (85)
Male	15 (26)	4 (15)
Relation – no. (%)		
Spouse	20 (33)	3 (11)
Daughter	23 (38)	15 (56)
Son	11 (18)	4 (15)
Other relative	6 (11)	5 (19)
Employment – no. (%)		
Full-time	19 (35)	6 (24)
Part-time	6 (11)	3 (12)
Retired	23 (42)	12 (48)
Homemaker	5 (9)	4 (16)
Not employed at this time	2 (4)	0
Live with – no. (%)		
Yes	23 (40)	6 (23)
No	35 (60)	20 (77)
Distance to residence – no. (%)		
16 to 30mins	30 (52)	13 (50)
30mins to 1 h	3 (5)	5 (19)
> 1 h	6 (10)	4 (15)
Family member lived with	19 (33)	4 (15)
Distance to hospital – no. (%)		
16 to 30mins	38 (66)	16 (62)
30mins to 1 h	14 (24)	7 (27)
> 1 h	6 (10)	3 (12)
Frequency of contact in person – no. (%)		
Once per week	10 (18)	1 (4)
1–3× per week	18 (32)	15 (60)
4–6× per week	5 (9)	2 (8)
Daily	11 (19)	4 (16)
More than once daily	13 (23)	3 (12)
Frequency of contact by phone – no. (%)		
Once per week	4 (9)	2 (8)
1–3× per week	14 (33)	8 (33)
4–6× per week	6 (14)	4 (17)
Daily	13 (30)	5 (21)
More than once daily	6 (14)	5 (21)
Self-Rated Health – no. (%)		
Excellent	12 (23)	10 (40)
Good	31 (59)	11 (44)
Fair/Poor	10 (19)	4 (16)

*Plus-minus values are means ± SD

**Percentages were rounded to the nearest whole number

#### Overall effectiveness

Differences between patient groups were examined in terms of intervention unit (IU) versus usual care (UC) unit, and whether or not patients received the HELP (Table 3). The point-prevalence rate of delirium at admission was higher on IU with six patients (10.9 %) presenting with a delirium, and one patient on UC (2.5 %). Delirium prevalence was the same in the two units at discharge; with one patient on IU (2.5 %) and one patient on UC (2.5 %). These results were the same when comparing patients who did, or did not, receive the HELP, i.e., only one patient in each group had a delirium at discharge.

**Table 3:** Study outcomes during hospitalization, according to study group (by intervention)

Study outcome	Intervention group (*N* = 35)^*, **^			Usual care group (*N* = 36)^*, **^			*P*-value		
	Pre	Post	Change	Pre	Post	Change	Pre	Post	Change
CAM – no. (%)									
Present	6 (17)	1 (3)	N/A	1 (2)	1 (2)	N/A	N/A	N/A	N/A
Absent	29 (83)	34 (97)		65 (98)	65 (98)				
MoCA									
Mean score ± SD	15.7 ± 5.6	17.8 ± 6.0	2.0 ± 4.0	17.4 ± 5.8	17.6 ± 6.1	0.1 ± 4.1	*P* = .172	*P* = .861	*P* = .049
Short-term memory and recall									
Mean score ± SD	0.9 ± 1.4	1.8 ± 1.7	0.8 ± 1.5	1.4 ± 1.6	1.3 ± 1.6	−0.1 ± 1.4	*P* = .198	*P* = .183	*P* = .006
FIM™ Total									
Mean score ± SD	54.1 ± 17.8	80.9 ± 25.1	25.9 ± 16.0	63.7 ± 16.6	85.0 ± 24.1	20.9 ± 17.6	*P* = .010	*P* = .446	*P* = .188
Cognitive									
Mean score ± SD	22.1 ± 8.5	25.5 ± 7.4	3.3 ± 5.6	25.7 ± 5.8	27.7 ± 9.1	2.1 ± 7.1	*P* = .016	*P* = .238	*P* = .406
Motor									
Mean score ± SD	32.2 ± 11.8	55.1 ± 20.7	22.5 ± 14.6	38.0 ± 13.2	57.3 ± 20.4	18.9 ± 15.6	*P* = .037	*P* = .620	*P* = .278
Rehabilitation Efficiency									
Mean score ± SD	N/A	0.5 ± 0.3	N/A	N/A	0.4 ± 0.4	N/A	N/A	*P* = .381	N/A
Number of Falls									
Mean ± SD	N/A	0.3 ± 0.6	N/A	N/A	0.3 ± 0.6	N/A	N/A	*P* = .785	N/A
Length of Stay – no. of days									
Mean ± SD	N/A	52.3 ± 22.8	N/A	N/A	59.2 ± 30.2	N/A	N/A	*P* = .244	N/A

*Plus-minus values are means ± SD

**Percentages were rounded to the nearest whole number

At baseline, IU patients scored lower on cognitive and functional measures than UC patients. Although differences in scores on the MoCA were not statistically significant, IU patients scored significantly lower on all components of the FIM™ upon admission. Most change and follow-up scores were not statistically significant between IU and UC patients, except on the short-term memory and recall component of the MoCA (0.6 vs.−0.3, *P* = .013). However, comparisons between those patients who received the HELP and those who did not showed an increased effect on all measures. The results indicated a trend toward greater improvement among patients who received the HELP compared to those who did not. These findings are presented in Table 3.

#### Potential implementation factors and successes of the HELP

Many perceived barriers to the implementation of the HELP were a consequence of limited resources. Specifically, the need for more volunteers was emphasized by participants. Staff members struggled to increase and maintain the volunteer count, as most volunteers were students with busy schedules. One staff member described the need for a different population of HELP volunteers:

*“We depend a lot right now on students and you know it’s the nature of the game; they’re only temporary. Their schedules are really crazy and so it’s really hard sometimes to keep them for any length of time. We would really like to try to recruit some more mature volunteers to help us have some stability.”*

There was a particular need for HELP volunteers during time gaps, such as evenings and weekends. One staff member explained how the organization of the HELP was dependent on the availability of the volunteers:

*“They’re having to organize the program around when the volunteers are available; not when we think we have our biggest need.”*

Another barrier to program implementation was the limited interaction and lack of collaboration between HELP volunteers and hospital staff. Communication was described as minimal by both participant groups. Volunteers discussed feelings of intimidation and being a nuisance. One HELP volunteer commented:

*“It’s kind of intimidating…you have to like practice what you’re going to say to them.”*

Some staff members did not see a need to communicate with the HELP volunteers on a clinical level; rather, the groups worked independently. Volunteers made sure not to interfere with the staff’s routine, which was viewed as the top priority. One staff member elaborated:

*“They’re pretty quiet…I find whenever I go in and if they’re in there, they sort of jump up right away and they’re willing to leave, which I think is in some ways good, right? Because if, as the professional, you’re there to do some intervention there with your patient, I guess it should take priority…but it’s almost like they’re too flighty…they don’t always need to just run out of the room.”*

Levels of knowledge about the HELP varied among participants. Volunteers considered the initial training sessions to be adequate but struggled to retain all of the information over time. Several clinical staff members were not trained on the HELP, and volunteer participants recognized that some staff was not well-informed about the program.

Knowledge about the HELP was also limited among patients. Although it was generally assumed that the program interventions were benefiting patients’ recovery process, many were unsure of the purpose and effectiveness of particular interventions. Patients perceived the interventions targeting immobility to be helpful, but considered those targeting cognitive impairment to be simple and juvenile. One patient said:

*“I think they are here to try to help, but I’m just not sure that what they do and things they ask are. Like, those little crossword puzzles that, they’re embarrassing simple I think. Even though it makes us stop and think for a moment.”*

While patients found the interventions targeting immobility to be most effective, those targeting cognitive impairment were highlighted by staff, volunteers, and caregivers. Many participants noticed a visible improvement, particularly in patients’ memory functioning. One spousal caregiver illustrated the changes he observed in his wife:

*“There was a comment earlier about delirium and trying to avoid it or minimize it, and I know my wife is a lot better now than she was when she first came in. Whether the program has helped, I’m not sure. If that’s a purpose, then maybe it has helped…she knows where she is and where she wants to go here, whereas at first she wasn’t too sure what town she was in.”*

The HELP was characterized by participants as a way to the fill patients’ time that is otherwise unoccupied. Many patients appreciated the HELP volunteers coming to see them when they were not able to participate in other activities. One patient said:

*“It’s helping you because someone has come along in a moment when you’re not doing anything and it fills up some of the time…if you can’t get down to do some of the things. You are just in your bed and somebody comes up, that’s great…that’s when I noticed it more, when I couldn’t do anything myself.”*

One caregiver suggested that providing patients with activities to engage them during their hospital stay should be a purpose of the HELP:

*“I think part of its job is taking the time that’s not utilized in other ways… I know maybe they have to work in physio sessions and one thing or another but…it’s so boring around here…so there’s lots of room for your volunteers, and not at the same time something else is going on.”*

Participants felt that the HELP also filled a gap when family and/or friends were not able to visit patients. The importance of the volunteers visiting patients whose loved ones were unavailable was frequently discussed. Many participants believed that the social aspect of the HELP is the most advantageous for the patients. One patient commented:

*“I find that the most people got out of this was the plain old visiting. They don’t need to be doing this or that, or exercise. They just need to see some friendly face and someone that’s going to listen to them.”*

Furthermore, it was noted that the HELP filled a gap when hospital staff members were unavailable to attend to patients due to lack of time. One caregiver provided an example:

*“The entrance nurses were very busy and then the first volunteer that came in and I think it was her first time too, her name was [HELP volunteer], she was very nice. She smiled and listened to [patient], and took her time; and even though everybody else was sort of rushing around because they were so busy…these people do an amazing job, but they are short staffed…the volunteers fill a gap there. They’re available; they’re kind.”*

### Linking the quantitative and qualitative results

At admission, delirium was more prevalent on IU than on UC, indicating that patients who were admitted with delirium were more likely to receive the HELP. The point-prevalence rate of delirium at discharge was equal between the two units, with one patient on IU and one on UC. These findings indicate a larger reduction in delirium prevalence from admission to discharge for patients who received the HELP compared to those who did not. Patient and caregiver participants suggested that those who were more cognitively and/or functionally impaired may have a greater need for the program than other patients. Caregivers noticed gains in the patients’ cognitive functioning as a result of the HELP, especially in patients with delirium at admission. Staff members also recognized that patients who were cognitively impaired at admission and participated in the HELP showed significant improvement by discharge.

Patients who received the HELP had a higher mean change score on the MoCA than that of patients who received usual care, despite having lower mean scores at admission. The mean change scores on the short-term memory and recall subscale of the MoCA were significantly higher for patients who received the HELP compared to usual care patients. These results showed a notable improvement in cognitive functioning, particularly short-term memory and recall, from admission to discharge for patients who received the HELP. Volunteers and staff members confirmed this finding; many noticed improvements in patients’ memory functioning when participating in the HELP and found the program interventions that target cognitive impairment to be most effective. Although participants agreed that no program component was ineffective, patients believed these interventions were too simple.

At admission, the average FIM™ score of patients who received the HELP was significantly lower than that of usual care patients, and remained lower at discharge. However, the average change score of the FIM™ was higher for patients who received the HELP than for those who did not. This finding indicates that patients who received the HELP were more impaired upon admission than UC patients, yet improved at a similar rate by discharge following participation in the program. Results of both the cognitive and motor subscales of the FIM™ showed similar trends. The qualitative data supported this finding. Caregivers recognized that patients’ ability to communicate with others, a component of the cognitive subscale of the FIM™, had improved since participation in the HELP. In regard to the motor subscale, patients who participated in the HELP identified the interventions targeting immobilization as most effectively helping them to recover. Staff participants also valued these interventions but recognized that the HELP volunteers were limited in their capacity to fully implement them.

## Discussion

This study examined the potential successes and barriers to the implementation of the HELP in a post-acute rehabilitation hospital setting. The results demonstrate that delirium is prevalent among older adults admitted to post-acute rehabilitation settings, and can potentially be prevented or managed through the HELP. The study findings also indicate that the HELP aids in the improvement of cognitive and functional outcomes as well as shortens length of stay for older patients in rehabilitation settings. Patients, caregivers, volunteers, and staff perceived the HELP to be effective in enhancing the rehabilitation process, which further strengthens the results of this study.

Point-prevalence rates indicated that patients with a present delirium at admission were more likely to receive the HELP. Some evidence suggests that patients with at least one risk factor for delirium could benefit from the program and targeting patients at an intermediate risk is an efficient and cost-effective approach [18]. Patient and caregiver participants believed that those patients with greater impairment were most likely to benefit from the interventions, and staff members specified that cognitively impaired patients who participated in the HELP noticeably improved during their stay. These findings indicate that where hospitals do not have the resources to enroll all eligible patients into the HELP, a barrier frequently mentioned by participants, the program should be offered to patients with higher levels of impairment.

The overall point-prevalence rate of delirium at admission (7 %) was less than that reported in an earlier study (23 %), in which clinical staff was specially trained to detect delirium in hip fracture patients in both rehabilitation hospitals and skilled nursing facilities [43]. This discrepancy could be explained by differences in patient characteristics, settings, or a lack of detection. The current study took place in a rehabilitation hospital where only 50 % of patients were admitted with hip fractures, and only those clinical staff on IU received training. Delirium rates may be higher upon admission to rehabilitation settings with improved detection through further staff training. Nonetheless, IU patients showed greater cognitive and functional impairment at admission than UC patients.

Some discrepancies between the results of this study and those of previous studies were also present at discharge. Patients who received the HELP showed a larger reduction in delirium prevalence (83 %) than that of earlier research, which showed that 64 % of patients with delirium symptoms at admission to post-acute facilities exhibited the same number, or more, one week later; and only 14 % of patients resolved their symptoms entirely [43]. Although there were differences between this earlier study and the current study (patient characteristics, settings, detection), it is arguable that the decrease in delirium prevalence among patients who received the HELP could be attributed to the program interventions.

Participants acknowledged the effectiveness of the HELP interventions. Staff, volunteers and caregivers perceived the orientation and therapeutic-activities protocols to be most effective whereas patients thought the early-mobilization protocol was most beneficial to their rehabilitation. These findings were supported by the quantitative results, with a trend toward greater improvement in both cognitive and functional outcomes, as well as shorter length of stay, among patients who received the HELP compared to those who did not. Patients who received the HELP showed greater impairment upon admission than UC patients, yet they achieved equal or more gains in less time. Therefore, the HELP interventions may aid in enhancing the recovery process of older patients in a post-acute rehabilitation setting.

The overall effectiveness of the HELP is highly dependent on the availability of resources, particularly the HELP volunteers. It was widely recognized among participants that volunteers are necessary for successful implementation of the program; however, several barriers were discussed, including the need for more HELP volunteers, their restricted capacity to fully implement certain interventions, and their limited interaction with the staff. Greater availability and increased capacity of HELP volunteers were recommended to enhance the effectiveness of the program. Both the HELP volunteers and staff described their interactions as minimal and problematic. Yet, staff members valued the role of the HELP volunteers in the rehabilitation process of the patients.

Patients, caregivers and staff members believed the HELP volunteers were competent, and appreciated their presence. Most participants felt that the social aspect of the HELP was most beneficial to patients. The HELP was perceived as a way to fill the gap when patients were unoccupied; when family and friends were unavailable; and when hospital staff was unavailable. Previous research found that the HELP enhanced both patient and family satisfaction with care [14], as well as work satisfaction among nurses and nurses’ aides [20]. The findings of this study were consistent with the existing literature. Participants believed that all participants generally benefited from the HELP; patients were engaged during their hospital stay, which supported the roles of caregivers, staff, and volunteers.

### Recommendations for further implementation

This study offers several recommendations for further implementation of the HELP in post-acute rehabilitation settings. First, staff participants discussed strategies to increase recruitment and retention of HELP volunteers. Similar to suggestions made in previous work [17], program leaders aimed to build partnerships with local community services to recruit a larger and more diverse population of volunteers. With an increased volunteer count, the HELP could expand to include more patients who may benefit from the program. A sufficient number of volunteers who have greater availability could also help to fill time gaps (i.e., evenings and weekends), which might aid in improving adherence to program interventions.

Adherence to HELP interventions was not only restricted by the need for more volunteers, but also the volunteers’ limited capacity to fully implement them. All participant groups recommended that the HELP volunteers receive continual education and training to increase capacity, as well as to retain knowledge and skills. Participants suggested that further education and training be offered to hospital staff in order to enhance the detection of delirium and general knowledge of the HELP. Moreover, education and training could be provided jointly to aid in strengthening relationships between staff members and HELP volunteers.

Both staff and HELP volunteers described their social interactions as minimal and ineffective. A lack of feedback was identified as a key barrier to collaboration between the two groups, and greater information exchange was considered necessary to build relationships and enhance the HELP. One study recommended that staff re-evaluate roles, goals and relationships with other disciplines to overcome the challenges of increasing collaboration and integrating the HELP volunteers into the care team [44]. Another study proposed regular staff meetings and team-building efforts to resolve interpersonal conflict [17]. The results of this study suggest that both groups participating in regular meetings and team-building efforts as well as thinking differently about their roles, goals, and relationships may help to increase feedback and collaboration between the HELP volunteers and hospital staff.

Lastly, participants offered recommendations for further implementation of the HELP interventions. Patients suggested that the interventions targeting cognitive impairment be modified, including alternative orientation questions and more challenging therapeutic activities. The importance of tailoring the HELP interventions according to the patients’ wants and needs was emphasized by volunteer participants. Volunteers also proposed collective HELP interventions, in addition to the standard individual interventions, to encourage participation and enhance opportunities for socialization among patients.

### Limitations

Limitations of this study should be considered. This work was conducted at a single site; therefore, the generalizability of the results may be limited. Randomization was not feasible however patients were assigned to units based on bed availability to minimize potential selection bias. At baseline, patients who received the HELP were more impaired than those who did not, indicating that patients displaying delirium symptoms were more likely to be enrolled in the program. As clinical staff was situated on each unit, it is plausible that staff located on IU were more sensitized to delirium and better able to detect it. The HELP volunteers were situated only on the intervention unit, which minimizes the threat of both co-intervention and contamination. The statistical significance of the study findings was restricted by a lack of statistical power due to a small sample size, as only 35 patients on the IU received the HELP. Separate analyses were conducted (by unit and by intervention) to strengthen the quantitative findings. Similar to previous research in acute care settings [44], it was difficult to demonstrate positive outcomes during the initial adoption of the HELP. Nevertheless, the results of this pilot study indicated positive trends of functional improvement for patients who received the HELP in a rehabilitation setting.

The small number of interview participants was another limitation of this study. Two of the three focus group interviews did not have a large number (6–12) of participants [33]. Individual interviews were conducted with those who were unable to attend the scheduled focus group interviews. Member checking was used during the qualitative data analysis. Three staff participants responded and agreed with the findings. No volunteer participants responded, and contact information was not provided by patients and caregivers. A triangulation approach [36] was used to integrate various data sources and methods (patient outcomes, self-reported questionnaires, and focus group and individual interviews) to help remove any biases that might emerge using only one method [35, 45]. Efforts to ensure methodological rigor were employed through auditing and regular consultations with colleagues.

## Conclusions

The findings of this pilot study could support future investigations of delirium and the HELP in post-acute rehabilitation hospitals and other non-traditional settings. Several successes and implementation factors of the HELP were identified. It is evident that delirium is prevalent in post-acute rehabilitation settings, and the HELP can be effective in improving the cognitive and physical functioning of patients at a moderate-to-high risk for delirium or those admitted with an existing delirium. The consistency between the quantitative and qualitative results further validates these findings. Results of this research suggest that the HELP may serve as a useful delirium management strategy in a post-acute rehabilitation hospital; however, additional research is warranted.

## Abbreviations

CAM, confusion assessment method; CCI, Charlson co-morbidity index; DSM-III-R, diagnostic and statistical manual of mental disorders, third edition, revised; ELS, Elder Life Specialist; FIM™, functional independence measure; HELP, Hospital Elder Life Program; IBM, International Business Machines; IU, intervention unit; MoCA, montreal cognitive assessment; OT, occupation therapy; PT, physical therapy; RISKPRO, patient and visitor safety reporting system; SD, standard deviation; SPSS, statistical package for the social sciences; UC, usual care
